# Utilization of Synthetic Near-Infrared Spectra via Generative Adversarial Network to Improve Wood Stiffness Prediction

**DOI:** 10.3390/s24061992

**Published:** 2024-03-21

**Authors:** Syed Danish Ali, Sameen Raut, Joseph Dahlen, Laurence Schimleck, Richard Bergman, Zhou Zhang, Vahid Nasir

**Affiliations:** 1 USDA Forest Service, Forest Products Laboratory, Madison, WI 53726, USA; syed.ali2@usda.gov (S.D.A.); richard.d.bergman@usda.gov (R.B.); 2 Department of Biological Systems Engineering, University of Wisconsin-Madison, Madison, WI 53706, USA; zzhang347@wisc.edu; 3 Warnell School of Forestry and Natural Resources, University of Georgia, Athens, GA 30602, USA; sameen.raut@uga.edu (S.R.); jdahlen@uga.edu (J.D.); 4 Department of Wood Science and Engineering, Oregon State University, Corvallis, OR 97331, USA; laurence.schimleck@oregonstate.edu

**Keywords:** convolutional neural network (CNN), data augmentation, deep learning, ensemble learning, generative adversarial network (GAN), gradient-boosting machines (GBMs), modulus of elasticity (MOE), wood materials

## Abstract

Near-infrared (NIR) spectroscopy is widely used as a nondestructive evaluation (NDE) tool for predicting wood properties. When deploying NIR models, one faces challenges in ensuring representative training data, which large datasets can mitigate but often at a significant cost. Machine learning and deep learning NIR models are at an even greater disadvantage because they typically require higher sample sizes for training. In this study, NIR spectra were collected to predict the modulus of elasticity (MOE) of southern pine lumber (training set = 573 samples, testing set = 145 samples). To account for the limited size of the training data, this study employed a generative adversarial network (GAN) to generate synthetic NIR spectra. The training dataset was fed into a GAN to generate 313, 573, and 1000 synthetic spectra. The original and enhanced datasets were used to train artificial neural networks (ANNs), convolutional neural networks (CNNs), and light gradient boosting machines (LGBMs) for MOE prediction. Overall, results showed that data augmentation using GAN improved the coefficient of determination (R^2^) by up to 7.02% and reduced the error of predictions by up to 4.29%. ANNs and CNNs benefited more from synthetic spectra than LGBMs, which only yielded slight improvement. All models showed optimal performance when 313 synthetic spectra were added to the original training data; further additions did not improve model performance because the quality of the datapoints generated by GAN beyond a certain threshold is poor, and one of the main reasons for this can be the size of the initial training data fed into the GAN. LGBMs showed superior performances than ANNs and CNNs on both the original and enhanced training datasets, which highlights the significance of selecting an appropriate machine learning or deep learning model for NIR spectral-data analysis. The results highlighted the positive impact of GAN on the predictive performance of models utilizing NIR spectroscopy as an NDE technique and monitoring tool for wood mechanical-property evaluation. Further studies should investigate the impact of the initial size of training data, the optimal number of generated synthetic spectra, and machine learning or deep learning models that could benefit more from data augmentation using GANs.

## 1. Introduction

The industrial scale characterization and quality control of wood and wood-based materials require the development of fast and reliable non-destructive evaluation (NDE) tools. Near-infrared (NIR) spectroscopy (wavelengths ranging from 800 to 2500 nm) is one of many NDE methods, and it has been widely investigated for wood-quality control and characterization purposes [1,2]. A variety of wood-related applications have been explored and include the prediction of chemical, physical, and mechanical properties, the classification and identification of wood species, and the performance of wood materials and timber structures under weathering and photodegradation [3,4,5,6]. Typically, NIR spectroscopy models utilize partial least squares (PLS) regression or principal components analysis (PCA) regression [7,8] and there has been considerable interest in using these approaches to predict mechanical properties (modulus of elasticity (MOE), modulus of rupture (MOR)) of wood [8]. A variety of species have been utilized for this purpose and include Norway spruce (*Picea abies*) [9,10], radiata pine (*Pinus radiata*) [11], loblolly pine (*Pinus taeda*) [12,13], longleaf pine (*Pinus palustris*) [14,15], hybrid larch (*Larix gmelinii* var. *japonica × Larix kaempferi*) [16,17], southern pine (*Pinus* spp.) [18], and eucalyptus species [19,20,21]. Typically, the number of samples utilized in these studies is relatively limited, with the studies by Thumm and Meder (2001) [11] that utilized 404 visible-NIR spectra and Dahlen et al. (2017) [18], who developed PLS regression models, with 718 NIR spectra amongst the largest.

Machine learning models are increasingly being used and can provide better predictive performance compared to traditional modeling frameworks because they are often more effective at capturing underlying patterns in the data. Datasets that are large and complex can benefit from machine learning models because they are able to model nonlinear relationships; moreover, some machine learning algorithms have built-in feature selection capabilities that can identify the most relevant features for predicting the response variable accurately [22,23]. Research on using machine learning and deep learning applied to NIR spectra for wood characterization and monitoring is relatively limited. Studies on small-sized NIR spectral datasets (sample sizes ranging from 172 to 480) showed artificial neural networks (ANNs) outperformed PLS regression models [24,25,26,27]. Specifically, Ayanleye et al. (2021) [26] used 240 samples to train ANN and neuro-fuzzy models to predict the MOE and MOR of western hemlock (*Tsuga heterophylla*) and Douglas-fir (*Pseudotsuga menziesii*) lumber. Similarly, Nasir et al. (2019) [28] studied the classification of thermally modified western hemlock wood, using 336 NIR spectra to train ANNs. Relatively small sample sizes pose a major obstacle for applying deep learning and machine learning to NIR spectroscopy in wood science and technology because Grinsztajn et al. (2022) [29] describe medium-sized tabular datasets as having approximately 10,000 samples in the calibration set for classification and regression problems. For wood science and engineering, no published literature comes close to these sample sizes. Thus, the performance of different machine learning and deep learning models when utilizing NIR spectral data may not provide representative data at the population level.

It is evident that the state-of-the-art models for predicting wood mechanical properties using NIR spectra are developed using small-sized datasets. One approach is to expand the number of collected NIR spectra by conducting larger-scale experiments, which is not a feasible solution in many cases due to the cost and challenges of testing additional samples. An alternative solution is to practice data augmentation, which involves generating synthetic data using generative models. Generative models are algorithms that generate new datapoints based on patterns and distributions of the datapoints in an existing dataset [30]. A specific type of generative models are generative adversarial networks (GANs), which are developed using deep neural networks and are widely used in many tasks including the generation of text, audio signals, spectral data, tabular data, time series data, and images [31,32,33,34,35,36,37]. Recently, GANs have been applied to spectral data for data augmentation to improve the performance of machine learning and deep learning models [38,39]. For example, Teng et al. (2019) [40] applied GAN to a small dataset (N = 500) collected by laser-induced breakdown spectroscopy and showed that classification accuracy improved when the original training data was enhanced using synthetic data from GAN. Applying different classifiers on 480 NIR spectra, Yang et al. (2021) [41] found that the model obtained by GAN using competitive learning yielded better generalization ability and improved classification accuracy when dealing with small-sized high-dimensional spectral data. In a related study, Zhang et al. (2022) [35] applied GAN to small-sized spectral data (N = 400) to predict the oil content of a single maize kernel using PLS and support vector regression. The results showed that GAN improved the predictive performances of the models and addressed the challenge associated with a limited number of training data. Similar observations were reported by Li et al. (2022) [42] when applying GAN to spectral data to classify the quality of wheat kernels using convolutional neural networks (CNNs), decision trees, and support vector machines (SVMs). Utilizing a different approach, Zheng et al. (2021) [43] applied bidirectional GAN to NIR spectra for an imbalanced multiclass classification task with insufficient samples within the class to address the challenges associated with both imbalanced classification and insufficient sample size.

Despite the proven effectiveness of GAN in different fields, the wood science and technology literature lacks examples of employing this approach, especially with respect to the prediction of wood’s mechanical properties. Further, comparative studies between different machine learning and deep learning models for analyzing NIR spectra collected from wood materials are limited. This study aimed to address these shortcomings using NIR spectra collected to predict the MOE of southern pine 2 × 4 lumber (N = 718). Models were built using ANN, CNN, and a light-gradient-boosting machine (LGBM), and the predictive performance of developed models was compared. The impact of the dataset size was analyzed by employing a GAN to generate synthetic spectra to enhance the training data with different sample sizes of synthetic spectra (N = 313, 573, 1000).

## 2. Materials and Methods

### 2.1. Materials

**Figure 1:**
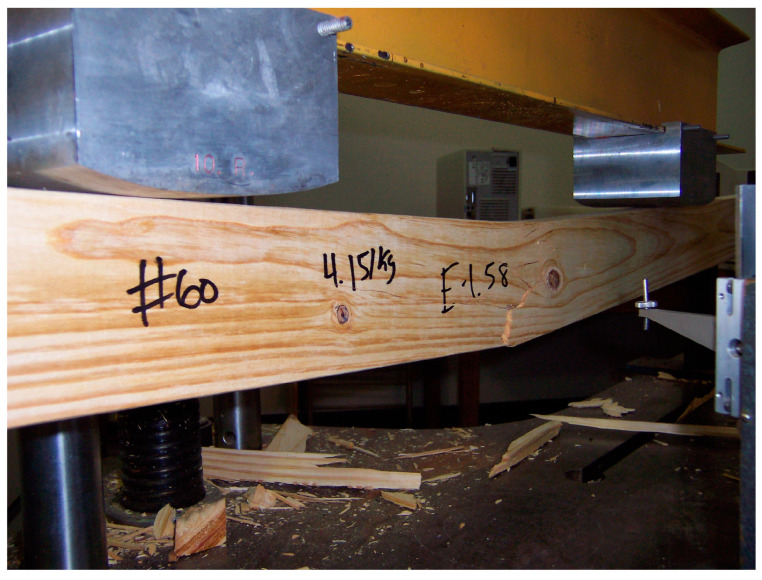
Destructive testing of a 2 × 4 lumber in edgewise bending as per ASTM standards.

Six packages of No. 2-grade, 2 × 4 sized, kiln-dried southern pine lumber with dimensions 38 mm × 89 mm × 2438 mm were obtained from commercial mills in Alabama, Arkansas, Georgia, Mississippi (2 mills sampled), and Texas [44]. From each package, 124 pieces of lumber (a total of 744) were destructively tested in edgewise bending as per ASTM standards using a Tinius Olsen deflectometer (Tinius Olsen Inc., Horsham, PA, USA) (Figure 1) [45]. The MOE of the lumber was determined from the measured deflection and load cell data [18]. Prior to each test, the MC of each piece was measured and the MOE values were adjusted to 15% MC [46]. After testing, a 38 mm radial × 89 mm tangential × 51 mm longitudinal block was cut from one end of each lumber piece using a radial arm saw; however, not every lumber piece yielded a usable block due to testing-related failures such as splitting in half or excessive cracking. Hence, a total of 718 blocks were available for spectral-data collection [18].

### 2.2. NIR Spectral Data Collection

**Figure 2:**
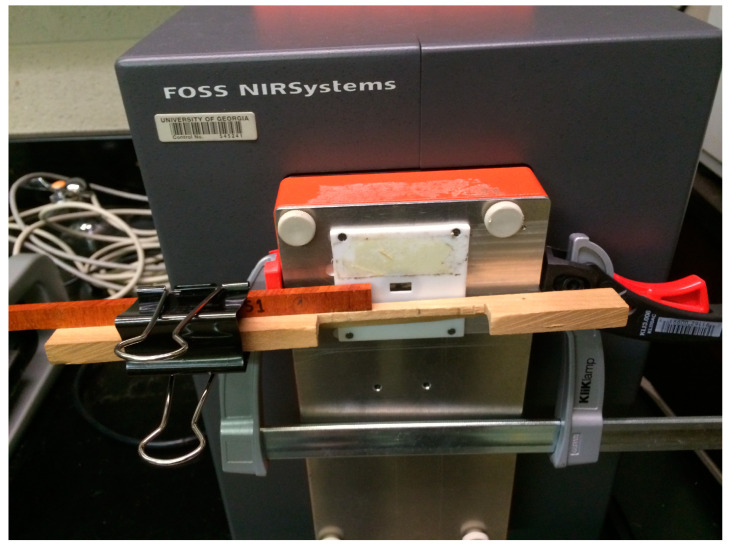
Acquisition of diffuse reflectance near-infrared spectral data from a wood sample on a FOSS NIRSystems Model 5000 scanning spectrophotometer.

Diffuse reflectance NIR spectra (1100–2500 nm at 2 nm intervals) were collected from one transverse face of each block using a FOSS NIRSystems Model 5000 scanning spectrophotometer (FOSS NIRSystems, Inc., Laurel, MD, USA) in a temperature (20 °C)- and humidity (40% RH)-controlled room (Figure 2). A ceramic standard was used as the instrument reference. A white Teflon mask with a square window of length 16.5 mm was fitted to the spectrophotometer to make sure a consistent sample area was scanned each time. For each block, two separate scans were taken, with each scan being an average of 32 readings. A single diffuse reflectance spectrum per block was then calculated by averaging the two separate scans. The diffuse reflectance (R) values were transformed to pseudo absorbance (A) using A = log10 (1/R). These absorbance values were subjected to a spectral pretreatment where second derivative spectra were calculated with the left and right gaps of four points using the Savitzky–Golay approach [47]. This resulted in a spectral dataset of 692 X-variables, which was used for data augmentation, and to fit the deep learning and machine learning models. The second derivative absorbance dataset was split (80/20) into training (N = 573) and testing (N = 145) datasets. The training dataset was used to train the deep learning and machine learning models and also for data augmentation using a GAN. The test dataset was kept independent and was only used for the final evaluation of the trained models.

### 2.3. Data Augmentation Using Generative Adversarial Network (GAN)

**Figure 3:**
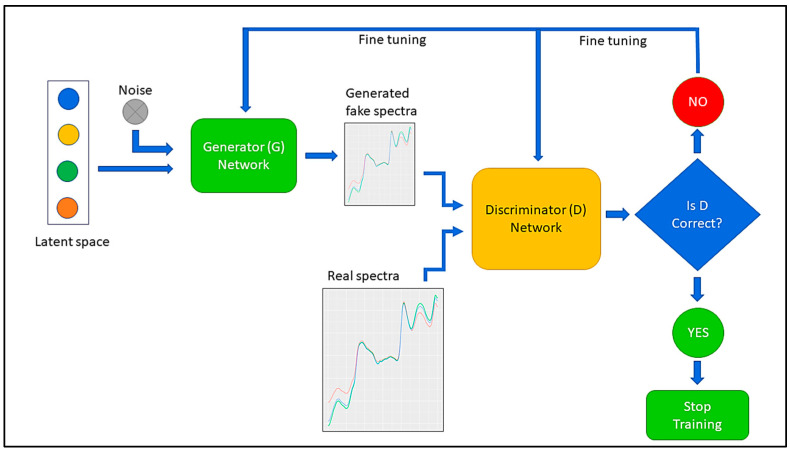
A schematic representation of the generative adversarial network (GAN) used in this study for generating fake NIR spectra.

To enhance the training dataset, deep learning-based synthetic spectra were generated using a GAN, an approach originally developed for image generation [31]. A GAN is a type of neural-network architecture consisting of two main components: a generator and a discriminator. The generator produces new data samples resembling the training data, while the discriminator is used to distinguish between real and fake data. The generator network commonly maps samples from a basic noise distribution to the target data distribution through fully connected layers. The generator and discriminator are trained together in a feedback loop, where the generator produces increasingly realistic samples, and the discriminator tries to correctly identify real from fake samples. This is continued until the data produced by the generator are indistinguishable from the real data according to the discriminator. A schematic representation of a GAN is shown in Figure 3. GANs have been successfully used for various tasks besides image generation, such as text generation [33], time-series-data generation [37], Raman-spectroscopy=data generation [48], hyperspectral-sample generation [49], and audio-signal generation [34]. Based on the same concept, NIR spectra were generated to augment the experimental dataset for training and improving the predictive performance of MOE models.

The GAN deployed here had three dense layers with 256, 512, and 692 neurons, respectively, in the generator. A rectified linear unit (ReLU) as an activation function was utilized for the first two layers, whereas the third layer used a ‘linear’ activation function. The first two discriminators of the dense layers consisted of 512 and 256 units with a ReLU as an activation, while the final output layer employed ‘sigmoid’ as an activation function. Adaptive moment estimation, ‘Adam’, was used for error backpropagation with a default learning rate (0.001). A batch size of 128 was used for training over 1000 epochs. Finally, to enhance the data available for training and the development of machine/deep learning models, sample sets that were roughly half (N = 313), equivalent (N = 573), and twice the size of the original data (N = 1000) were created. Therefore, the final enhanced training sets for the development of machine learning and deep learning models to predict the stiffness (MOE) of southern pine wood consisted of 886, 1146, and 1573 datapoints, respectively. Furthermore, during the training process with augmented datasets, the MOE was approximated by considering the distance between the fake spectra and the original spectra. To provide more clarity, we specifically calculated the minimum distance between the spectra of the original samples and the augmented samples. This enabled us to approximate the original MOE and effectively incorporate it into our analysis.

### 2.4. Model Fitting

The original and enhanced datasets were used to train ANNs, CNNs, and LGBMs for predicting lumber’s MOE. ANNs are biologically inspired mathematical models that can explain variations in almost any type of dataset with a good degree of accuracy. Therefore, these are one of the most widely used deep learning neural networks for regression and classification [27]. ANNs consist of input, hidden, and output layers, with the layers consisting of neurons that are interconnected by weighted links [50]. The number of hidden layers and the number of neurons in each of the layers are user defined, or they can be defined using an optimization algorithm. Increasing the number of hidden layers and the number of neurons in each of the hidden layers results in more complex models that tend to overfit to the training dataset, which translates to poor prediction capabilities on the independent test dataset. Hence, it is necessary to tune the model to an architecture with the number of hidden layers and neurons within each layer, along with learning rate, as hyperparameters that are more generalized to the type of dataset being studied.

**Figure 4:**
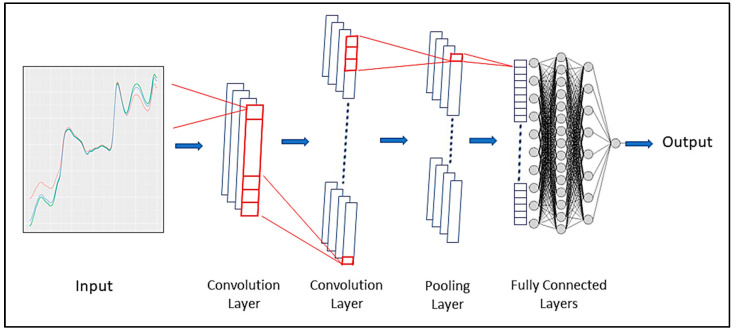
A schematic diagram of a convolutional neural network (CNN).

A CNN is a form of ANN that utilizes convolution, a specialized type of mathematical operation, in place of general matrix multiplication in a minimum of one of its layers [51,52]. A CNN consists of multiple layers such as convolution layers, pooling layers, activation layers, and fully connected layers that perform different operations on the input data (Figure 4). A convolution layer applies a set of filters to the input data, producing a set of feature maps that capture the local patterns in the data. A pooling layer reduces the size of the feature maps by applying a function such as maximum or average over a small region of the individual feature maps. An activation layer applies a nonlinear function to the feature maps, introducing nonlinearity and increasing the expressive power of the network. A fully connected layer connects every neuron in one layer to every neuron in the next layer, allowing the network to learn global features and perform classification or regression tasks. The convolution operation, weight sharing, and sparse connectivity in the CNN make it capable of processing images and other types of data with spatial structure. A convolution 1D (Conv1D) was employed to implement the CNN model for the prediction of the MOE using NIR spectral data in this study. When creating a model architecture, the hyperparameters that need tuning include the number of convolution layers, number of filters, size of filters, size of pooling, number of hidden layers, and number of neurons in each layer, together with the learning rate of the ‘Adam’ optimizer.

An effective, scalable, and optimized tree-based learning technique is the LGBM [53]. The LGBM technique employs a histogram-based decision-tree-learning technique that optimizes memory utilization and reduces communication overhead. Numerous applied machine learning tasks have used LGBM techniques because of its excellent predictive power, effectiveness, and capacity for handling complex datasets [27,54]. The algorithm has several key parameters that control overfitting, complexity, and the optimization process. The boosting type (boosting_type) parameter specifies the gradient-boosting decision tree as the boosting framework. This is the main algorithm behind the LGBM. The num_leaves parameter specifies the maximum number of leaves or terminal nodes per tree and affects model complexity and overfitting. The learning rate parameter specifies the shrinkage rate applied to each tree’s contribution and controls optimization speed and generalization. The feature_fraction parameter specifies the fraction of features sampled per tree to reduce overfitting. The bagging_fraction parameter specifies the fraction of data sampled per tree for stochastic bagging. The bagging_freq parameter specifies the frequency of bagging to perform stochastic bagging. The lambda_l1 and lambda_l2 parameters specify L1 and L2 regularization penalties on leaf weights to prevent overfitting. Other parameters include maximum tree depth, which impacts overfitting and complexity, and min_child_samples, which specify the minimum number of samples required in leaf nodes to prevent overfitting. Tuning these parameters is critical to maximizing the predictive performance of an LGBM model.

### 2.5. Hyperparameter Tuning, Model Training, and Evaluation

The optimization of hyperparameters has a significant impact on machine learning-model performance. In this study, the optimum hyperparameters for all the models were selected using the Python API Optuna. The Bayesian optimization-based sampler in Optuna, a tree-structured parzen estimator (TPE) which uses a probabilistic model to guide the search for hyperparameters, was used [55]. Using a tree-structured representation of the search space, the TPE simulates the probability distribution of the target function. This aids in the creation of fresh samples in regions that are most likely to produce the best results. Ten percent of the training data was used for the validation of the ANN and CNN models during the training process to evaluate model loss at every epoch, and with each iteration the weights of the models were updated. Early stopping was applied with a patience of 32, which would stop model training if the validation loss stayed constant or did not improve over 32 epochs. Finally, a batch size of 32 was employed. All the models were developed using Python version 3.9.13 (Python Software Foundation, https://www.python.org/ (accessed on 26 July 2023)), and the Keras library (https://keras.io/ required by TensorFlow version 2.10.0 (Google, Google Brain) (accessed on 26 July 2023)) was utilized for the development of the GAN, ANN, and CNN models. Figures were made in the R statistical programming environment version 4.2.2 (accessed on 1 March 2023) [56] using the RStudio interface version 2022.12.0 (accessed on 1 March 2023) [57] and the ggplot2 package [58].

The models were evaluated by comparing fit statistics such as the coefficient of determination (R^2^) and the root mean square error (RMSE) of predictions. R^2^ is a measure of how much variation in the dependent variable (MOE, in this study) is explained by the model using the independent variables (spectral data, in this study) as input. Values for R^2^ range from 0 to 1. An R^2^ value of 0 would mean that the model in question (either ANN, CNN, or LGBM, in this study) was not able to explain any variability in the MOE, whereas an R^2^ value of 1 would mean that the model was able to explain all of the variation in the MOE. Hence, for any model used in this study, it was desirable to have a higher R^2^ value because it suggests a better fit, as the model in question accounts for most of the variability in the MOE, using the spectral data provided as inputs. Also, a high R^2^ value suggests strong correlation between the independent and the dependent variables, but it does not confirm that the changes in independent variables cause the dependent variable to change. RMSE, which provides a measure of the model’s prediction error, is calculated by taking the square root of the average of the squared differences between the actual and the model-predicted values. A lower RMSE indicates a better model fit and suggests a higher accuracy of the model in making predictions.

## 3. Results and Discussions

**Figure 5:**
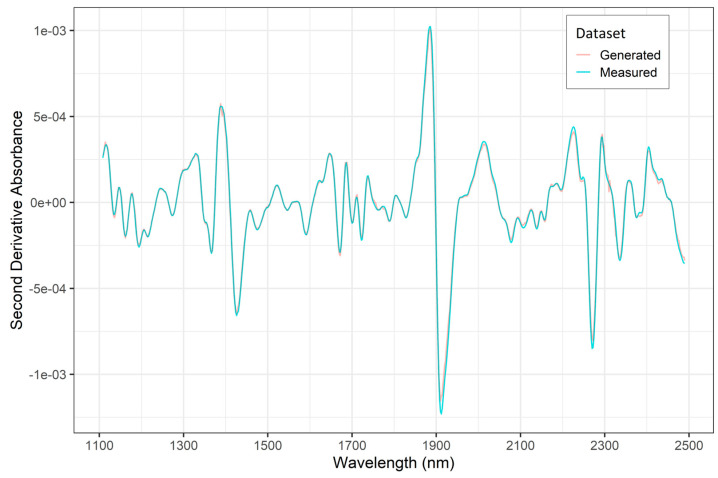
Mean second derivate NIR spectra of the measured (N = 573) and the generated (N = 313) training datasets.

**Figure 6:**
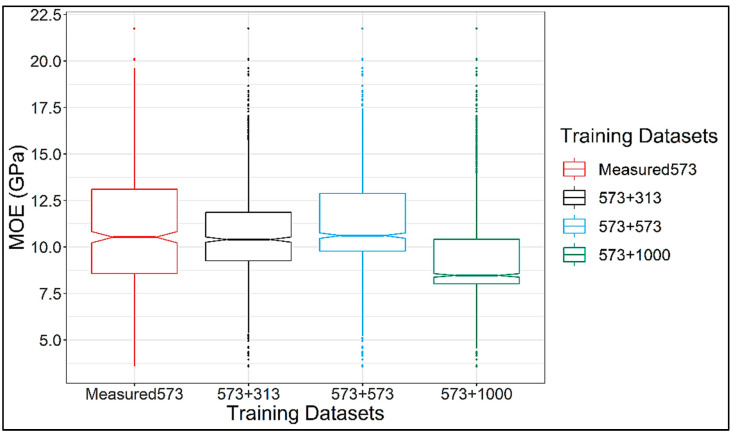
Boxplots showing the distribution of the MOE in measured and enhanced training datasets.

A plot showing the mean second derivative NIR spectra of the measured training dataset (N = 573) and the generated training datapoints (N = 313) is shown in Figure 5. The two spectra are in very close agreement with each other, highlighting that the GAN was able to generate very realistic spectral data to augment the original/measured training datasets. Summary statistics of the MOE (GPa) values in the measured training and testing datasets along with the three enhanced datasets is provided in Table 1. A visual representation of the MOE distribution in these four training datasets is given in Figure 6. The boxplots show that the interquartile range of MOE values in the enhanced datasets is not as wide as that of the measured dataset, which means that the middle 50% of the data in the enhanced datasets have a narrow spread compared to the measured data. It is necessary to note that the datasets shown in Figure 6 are in an ascending order of size, and the shape of the boxplots give an idea of where the GAN was adding more datapoints to augment the original training dataset.

Training and test results for the three types of models used in this study on measured and three enhanced datasets are provided in Table 2. Overall, the LGBM models outperformed both the ANN and CNN models with better prediction statistics on the independent test dataset. The LGBM models also had superior prediction capabilities when trained on enhanced datasets as compared to their ANN and CNN counterparts. ANN models had the worst prediction statistics across all four training datasets with the performance of CNN models between the ANN and LGBM models. When trained on the measured/original training dataset, the LGBM model achieved a test R^2^ of 0.61, which is 10.90% and 7.02% higher than that obtained by the ANN (R^2^ = 0.55) and the CNN (R^2^ = 0.57), respectively. The test RMSE for this LGBM model was 2.22 GPa; a 7.88% and 4.72% improvement over the RMSE values for the ANN (2.41 GPa) and CNN models (2.33 GPa), respectively.

**Figure 7:**
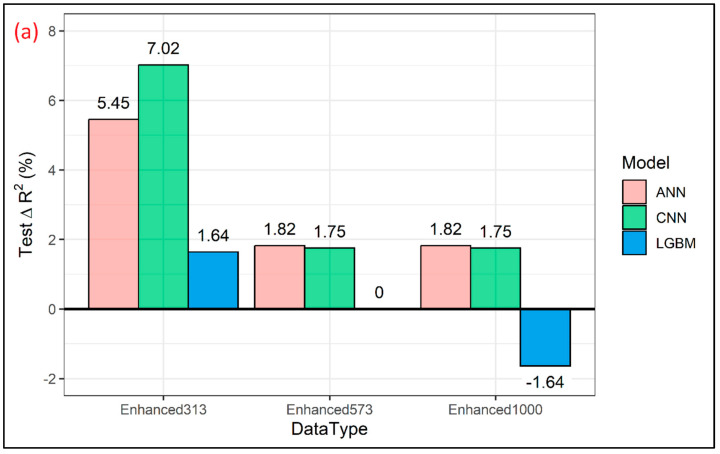

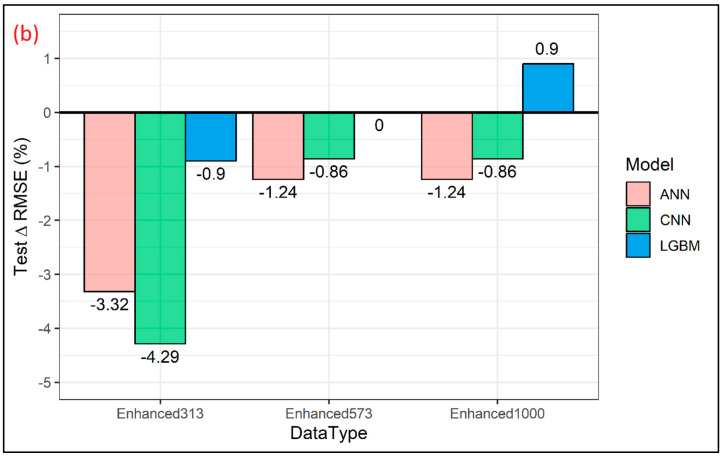
Bar plots showing percentage changes in test R^2^ (**a**) and the RMSE (**b**) for each of the enhanced datasets reported in relation to those from the models trained on the original dataset (N = 573).

**Figure 8:**
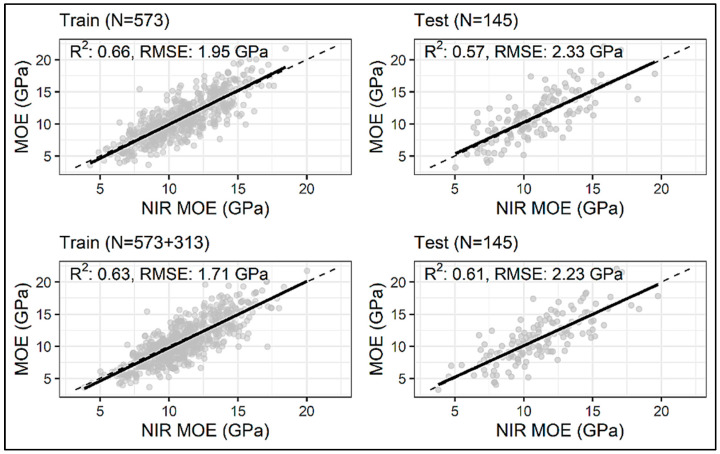
Actual vs. NIR-predicted test dataset MOE values from the CNN model trained on the original/measured dataset (N = 573) and the dataset enhanced by 313 datapoints (N = 573 + 313). The dashed black line denotes the line of equivalence.

**Figure 9:**
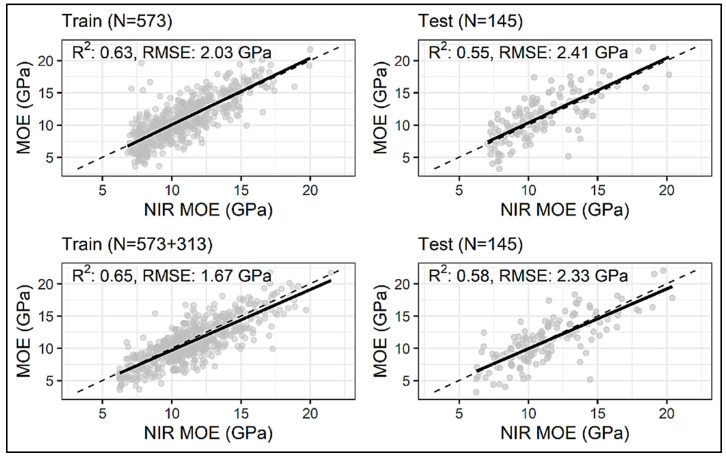
Actual vs. NIR-predicted test dataset MOE values from the ANN model trained on the original/measured dataset (N = 573) and the dataset enhanced by 313 datapoints (N = 573 + 313). The dashed black line denotes the line of equivalence.

**Figure 10:**
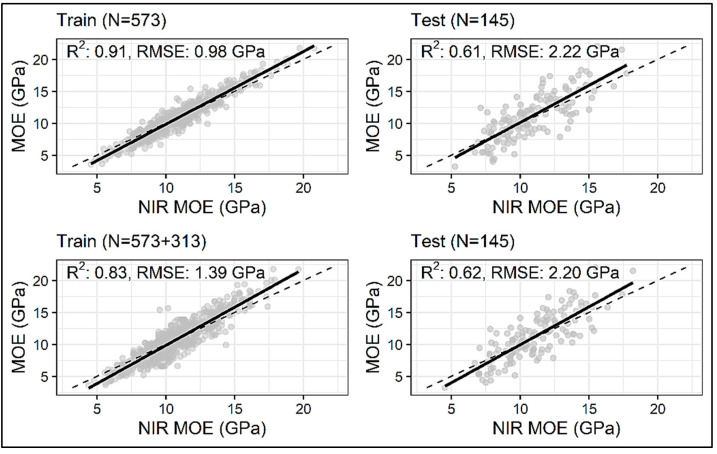
Actual vs. NIR-predicted test dataset MOE values from the LGBM model trained on the original/measured dataset (N = 573) and the dataset enhanced by 313 datapoints (N = 573 + 313). The dashed black line denotes the line of equivalence.

All models had the best prediction results with training on the dataset enhanced by 313 datapoints (Table 2). The biggest improvement in prediction performance is reported for the CNN model, which experienced an improvement in test R^2^ and RMSE by 7.02% and 4.29%, respectively (Figure 7). The first layer of this Conv1D model consisted of 256 filters, each with a length of 3, and a ReLU-activation function. The second Conv1D layer had 47 filters each with a length of 2, followed by the MaxPooling1D layer with a pool size of 2. The extracted features from the convolution and maxpooling layers were flattened and fed into the dense layers; three fully connected layers were employed, with 23, 81, and 18 neurons, respectively, with the ReLU as an activation function and the L2 regularization of 0.00001. The fully connected layers were followed by another dense layer with 32 neurons and a ReLU as an activation function. ‘Adam’ was used as an optimizer with a learning rate of 4.11 × 10^−5^. This model yielded a test R^2^ of 0.61 and a test RMSE of 2.23 GPa (Figure 8). The ANN model had an improvement on the test R^2^ by 5.45% and RMSE by 3.32% (Figure 7) when 313 synthetic spectra generated using a GAN were added to the original training dataset. This particular model consisted of four hidden layers with 23, 81, 14, and 108 neurons in each of the layers, and the activation function used was a ReLU. An ‘Adam’ optimizer with a learning rate of 0.018 was used for error backpropagation. This model gave a test R^2^ of 0.58 and a test RMSE of 2.33 GPa (Figure 9). The LGBM model showed the least improvement in prediction statistics on the test dataset (R^2^ improved by 1.64% and RMSE by 0.90% (Figure 7)). This slight improvement again resulted in LGBM outperforming the ANN and CNN, yielding a test R^2^ of 0.62 and an RMSE of 2.20 GPa (Figure 10). The optimal LGBM model used the traditional gradient-boosting decision tree (‘gbdt’) as the boosting type. The optimum learning rate, number of leaves, feature fraction, and the bagging fraction were found to be 0.066, 30, 0.685, and 0.718, respectively. Also, the bagging frequency, lambda_l1, and lambda_l2 parameters, maximum tree depth, and min_child_samples were set to 1, 0.015, 1.709 × 10^−6^, 3, and 12, respectively.

Adding a further number of generated datapoints, 573 and then 1000, did not further improve the prediction capabilities of the models (Table 2). The ANN model achieved identical prediction statistics for datasets enhanced by 573 and 1000 GAN-generated datapoints (R^2^ = 0.56 and RMSE = 2.38 GPa), which is an improvement in R^2^ by 1.82% and RMSE by 1.24% compared to just training on the original dataset (Figure 7). A similar case for the CNN model was observed as it achieved identical prediction statistics for datasets enhanced by 573 and 1000 GAN-generated datapoints (R^2^ = 0.58 and RMSE = 2.31 GPa), which is an improvement in R^2^ by 1.75% and RMSE by 0.86% compared to just training on the original dataset with 573 measured datapoints. Effectively, the deep learning models had an initial boost in prediction performance when 313 GAN-generated datapoints were added, but as a higher number of synthetic datapoints were included, the prediction performance of those models decreased but still outperformed the model trained on the original dataset. With LGBMs, adding 573 GAN-generated datapoints did not improve model training at all, as the test R^2^ and the RMSE remained identical when compared to model training done on measured data alone (Table 2). Further adding generated datapoints (1000 datapoints) showed a reduction in test R^2^ by 1.64% and an increase in the RMSE by 0.90% when compared to model training done on measured data alone (Figure 7). This showed that adding a greater number of synthetic datapoints for LGBM models is not advised beyond a certain point.

Data augmentation effectively contributed to enhancing the performance of ANNs and CNNs, whereas LGBMs had only a slight improvement using GANs. However, the predictive performance of LGBMs even without any generation of synthetic spectra was better than the ANN and almost equal to the optimal performance achieved by the CNN following data augmentation. The fact that the initial LGBM model on the original dataset performed better than the GAN-enhanced dataset for the ANN and CNN emphasizes the importance of choosing the correct type of machine learning or deep learning model for analyzing NIR spectral data. Training on tabular data, the general superior performance of models based on the gradient-boosted decision tree over deep learning methods has been reported, specifically where machine learning models outperformed deep learning models in regression [59]. Nasir et al. (2023) [27] showed that tree-based gradient-boosting machines such as LGBMs, XGBoost, and TreeNet outperformed the ANN and CNN models when predicting fiber properties using NIR spectral data (with and without applying PCA). Thus, one might speculate that the LGBM model was so robust on the original training dataset that it did not experience significant improvement in its performance by changing the size of the training data. However, the ANN and CNN could capture more complex relationships between the NIR spectra and the wood’s mechanical properties (here, MOE) when a larger training dataset was used.

Another important factor affecting the performance is the hyperparameter tuning performed on all the developed LGBM, CNN, and ANN models using the Bayesian optimization-based sampler Optuna TPE. In a study by Li et al. (2022) [42] on using GAN for improving the discrimination of unsound wheat kernels, the authors applied GAN to NIR spectral data to improve the classification accuracy of CNN, SVM, and decision-tree models. Their study does not mention performing hyperparameter tuning on the CNN and decision tree when changing the size of the training data. For the CNN model, Li et al. (2022) [42] showed an increase in classification accuracy (from 79.17% to 96.67%) as a result of a GAN, where a CNN could have learned more features as the number of samples increased. However, the rate of improvement could have been different if the hyperparameters were tuned every time the training dataset was changed by increasing the number of synthetic data generated by the GAN. In other words, one might hypothesize that the role of GANs in improving the predictive performance of NIR spectra-based models depends on the type of the selected model (machine learning vs. deep learning), subsequent hyperparameter tuning, as well as the model structure.

The performance of the models in this study varied depending on the quantity of synthetic samples added. Adding a larger number of synthetic samples (573 or 1000) did not consistently improve prediction performance, emphasizing the importance of finding an optimal balance between the original and synthetic data. Additionally, the quality and quantity of the experimental data used to train the model strongly affects the quality of the synthetic data produced by GANs [36]. In this study, all models yielded their best performances when they had an enhanced (N = 573 + 313) training dataset, and adding additional synthetic NIR spectra to the training dataset did not further improve the model. Zhang et al. (2022) [35] reported that PLS and support vector regression models achieved better statistics when predicting the oil content of a single maize kernel of one variety as they sequentially added increasing amounts of data generated by a GAN of up to 30 datapoints (in increments of 10). Sequentially adding datapoints beyond 30 resulted in decreasing model prediction performance, similar to what we observed. However, Li et al. (2022) [42] showed that CNN, SVM, and decision-tree models reached their optimal performance with training datasets having different sample sizes. They also showed that adding some synthetic NIR spectra could result in a local optimum performance of the model, whereas adding more synthetic NIR spectra may yield a global optimum. Therefore, finding the optimal size of the training dataset resulting in the best model performance is a crucial task when using GANs. Finally, our NIR spectral data was collected on one transverse face of the tested lumber without considering the impact of defects such as knots on its MOE. An issue with this approach in the NIR data may not accurately represent the lumber pieces whose mechanical properties are significantly affected by the defects [60]. This could impose a limitation on the maximum predictive performance that a machine learning or deep learning model could yield. Using hyperspectral imaging systems and scanning a greater area of the lumber studied may result in more representative NIR spectra for predicting the MOE of full-size lumber.

Using a GAN to augment datasets for the purpose of training models to enable them to have better predictive performances for MOE has practical applications. Accurate predictions of MOE enable structural engineers to select the right materials that meet the specific requirements for stiffness. This is particularly important in the present context, given that the demand and popularity of mass timber buildings have reached an unprecedented level. Using materials with the right stiffness at the right locations during construction will not only make the structure safer but cheaper to construct, which is important to promote wood as a sustainable construction material.

## 4. Conclusions

This study highlights the potential of NIR spectroscopy data augmentation using GANs to enhance the prediction of southern pine lumber’s MOE. A machine learning-based model for predicting wood’s MOE using NIR spectral data was proposed. A deep learning-based data-augmentation technique (a GAN) was utilized for enhancing the number of limited experimental training data samples, which improved feature-representation learning from NIR spectra and the predictability of machine learning methods. To achieve high predictability and solve the constraint of a small set of experimental data for training machine learning models, a GAN-based model for data augmentation was proposed and three machine/deep learning-based frameworks were developed using ANNs, CNNs, and LGBMs for the prediction of lumber’s MOE. The synthetic NIR spectra were in close agreement with those obtained from the original experimental samples. The results indicated that LGBMs achieved superior prediction performance compared to ANNs and CNNs, even though the latter both benefited more from data augmentation. This study signifies that using GAN for data augmentation is an effective approach to address the limitation of small experimental sample sizes in training machine/deep learning models applied to wood science and engineering problems. These findings contribute to the advancement of non-destructive testing methods for wood quality assessment and have practical implications for optimizing lumber-production processes. Future research could explore the application of other data augmentation techniques. Analyzing machine learning or deep learning models that could potentially benefit more from GANs and finding the optimal number of synthetic NIR spectra to be added to a training dataset should be further investigated. More emphasis should be placed on the impact of size and distribution of the initial training data on the performance of GAN. Finally, the use of similar techniques should be explored to predict different properties of wood and wood products.

## Figures and Tables

**Table 1:** Summary statistics of MOE values in the original training (original and enhanced) and testing datasets.

Dataset	Min	1st Quartile	Median	Mean	3rd Quartile	Max
Training dataset MOE (GPa) (N = 573)	3.577	8.579	10.529	10.920	13.112	21.761
Test dataset MOE (GPa) (N = 145)	3.211	8.771	10.913	11.129	13.600	22.040
Enhanced Training dataset MOE (GPa) (N = 573 + 313)	3.577	9.259	10.400	10.706	11.864	21.761
Enhanced Training dataset MOE (GPa) (N = 573 + 573)	3.577	9.779	10.614	10.897	12.886	21.761
Enhanced Training dataset MOE (GPa) (N = 573 + 1000)	3.577	8.028	8.473	9.608	10.415	21.761

**Table 2:** Training and test results of the three models fitted to the original and enhanced datasets of different sizes.

Model	Property	Train R^2^	Test R^2^	Train RMSE (GPa)	Test RMSE (GPa)
ANN	MOE Original (N = 573)	0.63	0.55	2.03	2.41
	MOE Enhanced (N = 573 + 313)	0.65	0.58	1.67	2.33
	MOE Enhanced (N = 573 + 573)	0.68	0.56	1.56	2.38
	MOE Enhanced (N = 573 + 1000)	0.66	0.56	1.48	2.38
CNN	MOE Original (N = 573)	0.66	0.57	1.95	2.33
	MOE Enhanced (N = 573 + 313)	0.63	0.61	1.71	2.23
	MOE Enhanced (N = 573 + 573)	0.58	0.58	1.76	2.31
	MOE Enhanced (N = 573 + 1000)	0.65	0.58	1.50	2.31
LGBM	MOE Original (N = 573)	0.91	0.61	0.98	2.22
	MOE Enhanced (N = 573 + 313)	0.83	0.62	1.39	2.20
	MOE Enhanced (N = 573 + 573)	0.72	0.61	1.77	2.22
	MOE Enhanced (N = 573 + 1000)	0.74	0.60	1.29	2.24

## Data Availability

Not available as data are part of ongoing research by authors.

## References

[1] Schimleck L., Dahlen J., Apiolaza L.A., Downes G., Emms G., Evans R., Moore J., Pâques L., Van den Bulcke J., Wang X. (2019). Non-destructive evaluation techniques and what they tell us about wood property variation. Forests.

[2] Nasir V., Ayanleye S., Kazemirad S., Sassani F., Adamopoulos S. (2022). Acoustic emission monitoring of wood materials and timber structures: A critical review. Constr. Build. Mater..

[3] Tsuchikawa S., Kobori H. (2015). A review of recent application of near infrared spectroscopy to wood science and technology. J. Wood Sci..

[4] Willems W., Lykidis C., Altgen M., Clauder L. (2015). Quality control methods for thermally modified wood: COST action FP0904 2010–2014: Thermo-hydro-mechanical wood behaviour and processing. Holzforschung.

[5] Tsuchikawa S. (2007). A review of recent near infrared research for wood and paper. Appl. Spectrosc. Rev..

[6] Tsuchikawa S., Schwanninger M. (2013). A review of recent near-infrared research for wood and paper (Part 2). Appl. Spectrosc. Rev..

[7] Sandak J., Sandak A., Meder R. (2016). Assessing trees, wood and derived products with near infrared spectroscopy: Hints and tips. J. Near. Infrared Spectrosc..

[8] Schimleck L.R., Matos J.L.M., Trianoski R., Prata J.G. (2018). Comparison of methods for estimating mechanical properties of wood by NIR spectroscopy. J. Spectrosc..

[9] Hoffmeyer P., Pedersen J. (1995). Evaluation of density and strength of Norway spruce wood by near-infrared reflectance spectroscopy. Holz Roh Werkst.

[10] Haartveit E.Y., Flæte P.O. (2006). Rapid prediction of basic wood properties by near infrared spectroscopy. N. Z. J. For. Sci..

[11] Thumm A., Meder R. (2001). Stiffness prediction of radiata pine clearwood test pieces using near infrared spectroscopy. J. Near Infrared Spectrosc..

[12] Kelley S.S., Rials T.G., Snell R., Groom L.H., Sluiter A. (2004). Use of near infrared spectroscopy to measure the chemical and mechanical properties of solid wood. Wood Sci. Technol..

[13] Schimleck L.R., Jones P.D., Clark A., Daniels R.F., Peter G.F. (2005). Near infrared spectroscopy for the nondestructive estimation of clear wood properties of *Pinus taeda* L. from the southern United States. For. Prod. J..

[14] Via B.K., So C.L., Shupe T.F., Eckhardt L.G., Stine M., Groom L.H. (2005). Prediction of wood mechanical and chemical properties in the presence and absence of blue stain using two near infrared instruments. J. Near Infrared Spectrosc..

[15] Via B.K., Shupe T.F., Groom L.H., Stine M., So C.L. (2003). Multivariate modelling of density, strength and stiffness from near infrared spectra for mature, juvenile and pith wood of longleaf pine (*Pinus palustris*). J. Near Infrared Spectrosc..

[16] Fujimoto T., Yamamoto H., Tsuchikawa S. (2007). Estimation of wood stiffness and strength properties of hybrid larch by near-infrared spectroscopy. Appl. Spectrosc..

[17] Fujimoto T., Kurata Y., Matsumoto K., Tsuchikawa S. (2008). Application of near infrared spectroscopy for estimating wood mechanical properties of small clear and full length lumber specimens. J. Near Infrared Spectrosc..

[18] Dahlen J., Diaz I., Schimleck L., Jones P.D. (2017). Near-infrared spectroscopy prediction of southern pine No. 2 lumber physical and mechanical properties. Wood Sci. Technol..

[19] Schimleck L.R., Evans R., Ilic J. (2001). Estimation of Eucalyptus delegatensis wood properties by near infrared spectroscopy. Can. J. For. Res..

[20] Kothiyal V., Raturi A. (2011). Estimating mechanical properties and specific gravity for five-year-old Eucalyptus tereticornis having broad moisture content range by NIR spectroscopy. Holzforschung.

[21] Zhao R., Huo X., Zhang L. (2009). Estimation of modulus of elasticity of Eucalyptus pellita wood by near infrared spectroscopy. Spectrosc. Spectr. Anal..

[22] Miotto R., Wang F., Wang S., Jiang X., Dudley J.T. (2018). Deep learning for healthcare: Review, opportunities and challenges. Brief. Bioinform..

[23] Wang J., Ma Y., Zhang L., Gao R.X., Wu D. (2018). Deep learning for smart manufacturing: Methods and applications. J. Manuf. Syst..

[24] Watanabe K., Kobayashi I., Matsushita Y., Saito S., Kuroda N., Noshiro S. (2014). Application of near-infrared spectroscopy for evaluation of drying stress on lumber surface: A comparison of artificial neural networks and partial least squares regression. Dry. Technol..

[25] Costa L.R., Tonoli G.H.D., Milagres F.R., Hein P.R.G. (2019). Artificial neural network and partial least square regressions for rapid estimation of cellulose pulp dryness based on near infrared spectroscopic data. Carbohydr. Polym..

[26] Ayanleye S., Nasir V., Avramidis S., Cool J. (2021). Effect of wood surface roughness on prediction of structural timber properties by infrared spectroscopy using ANFIS, ANN and PLS regression. Eur. J. Wood Wood Prod..

[27] Nasir V., Ali S.D., Mohammadpanah A., Raut S., Nabavi M., Dahlen J., Schimleck L. (2023). Fiber quality prediction using NIR spectral data: Tree-based ensemble learning vs. deep neural networks. Wood Fiber Sci..

[28] Nasir V., Nourian S., Zhou Z., Rahimi S., Avramidis S., Cool J. (2019). Classification and characterization of thermally modified timber using visible and near-infrared spectroscopy and artificial neural networks: A comparative study on the performance of different NDE methods and ANNs. Wood Sci. Technol..

[29] Grinsztajn L., Oyallon E., Varoquaux G., Koyejo S., Mohamed S., Agarwal A., Belgrave D., Cho K., Oh A. (2022). Why do tree-based models still outperform deep learning on typical tabular data?. Advances in Neural Information Processing Systems.

[30] Lamb A. (2021). A brief introduction to generative models. arXiv.

[31] Goodfellow I., Pouget-Abadie J., Mirza M., Xu B., Warde-Farley D., Ozair S., Courville A., Bengio Y., Ghahramani Z., Welling M., Cortes C., Lawrence N., Weinberger K.Q. (2014). Generative adversarial nets. Advances in Neural Information Processing Systems.

[32] Nagasawa T., Sato T., Nambu I., Wada Y. (2020). fNIRS-GANs: Data augmentation using generative adversarial networks for classifying motor tasks from functional near-infrared spectroscopy. J. Neural Eng..

[33] Zhang Y., Gan Z., Fan K., Chen Z., Henao R., Shen D., Carin L., Precup D., Teh Y.W. (2017). Adversarial feature matching for text generation. Proceedings of the 34th International Conference on Machine Learning.

[34] Engel J., Agrawal K.K., Chen S., Gulrajani I., Donahue C., Roberts A. (2019). GANSynth: Adversarial neural audio synthesis. arXiv.

[35] Zhang L., Wang Y., Wei Y., An D. (2022). Near-infrared hyperspectral imaging technology combined with deep convolutional generative adversarial network to predict oil content of single maize kernel. Food Chem..

[36] Little C., Elliot M., Allmendinger R., Samani S.S. (2021). Generative adversarial networks for synthetic data generation: A comparative study. arXiv.

[37] Smith K.E., Smith A.O. (2020). Conditional GAN for timeseries generation. arXiv.

[38] Hu J., Yang H., Zhao G., Zhou R. (2022). Research on online rapid sorting method of waste textiles based on near-infrared spectroscopy and generative adversity network. Comput. Intell. Neurosci..

[39] Zhu D., Xu L., Chen X., Yuan L., Huang G., Li L., Chen X., Shi W. (2020). Synthetic spectra generated by boundary equilibrium generative adversarial networks and their applications with consensus algorithms. Opt. Express.

[40] Teng G.E., Wang Q.Q., Kong J.L., Dong L.Q., Cui X.T., Liu W.W., Wei K., Xiangli W.T. (2019). Extending the spectral database of laser-induced breakdown spectroscopy with generative adversarial nets. Opt. Express.

[41] Yang B., Chen C., Chen F., Chen C., Tang J., Gao R., Lv X. (2021). Identification of cumin and fennel from different regions based on generative adversarial networks and near infrared spectroscopy. Spectrochim. Acta—Part A Mol. Biomol. Spectrosc..

[42] Li H., Zhang L., Sun H., Rao Z., Ji H. (2022). Discrimination of unsound wheat kernels based on deep convolutional generative adversarial network and near-infrared hyperspectral imaging technology. Spectrochim. Acta—Part A Mol. Biomol. Spectrosc..

[43] Zheng A., Yang H., Pan X., Yin L., Feng Y. (2021). Identification of multi-class drugs based on near infrared spectroscopy and bidirectional generative adversarial networks. Sensors.

[44] Dahlen J., Jones P.D., Seale R.D., Shmulsky R. (2013). Mill variation in bending strength and stiffness of in-grade southern pine No. 2 2 × 4 lumber. Wood Sci. Technol..

[45] (2015). Standard Test Methods of Static Tests of Lumber in Structural Sizes.

[46] (2016). Standard Practice for Establishing Allowable Properties for Visually-Graded Dimension Lumber from In-Grade Tests of Full-Size Specimens.

[47] Savitzky A., Golay M.J. (1964). Smoothing and differentiation of data by simplified least squares procedures. Anal. Chem..

[48] Wu M., Wang S., Pan S., Terentis A.C., Strasswimmer J., Zhu X. (2021). Deep learning data augmentation for Raman spectroscopy cancer tissue classification. Sci. Rep..

[49] Liu X., Qiao Y., Xiong Y., Cai Z., Liu P. (2020). Cascade conditional generative adversarial nets for spatial-spectral hyperspectral sample generation. Sci. China Inf. Sci..

[50] Fernandes A., Lousada J., Morais J., Xavier J., Pereira J., Melo-Pinto P. (2013). Comparison between neural networks and partial least squares for intra-growth ring wood density measurement with hyperspectral imaging. Comput. Electron. Agric..

[51] Gu J., Wang Z., Kuen J., Ma L., Shahroudy A., Shuai B., Liu T., Wang X., Wang G., Cai J. (2018). Recent advances in convolutional neural networks. Pattern Recognit..

[52] Lecun Y., Bengio Y., Hinton G. (2015). Deep learning. Nature.

[53] Ke G., Meng Q., Finley T., Wang T., Chen W., Ma W., Ye Q., Liu T.-Y., Guyon I., Von Luxburg U., Bengio S., Wallach H., Fergus R., Vishwanathan S., Garnett R. (2017). LightGBM: A highly efficient gradient boosting decision tree. Advances in Neural Information Processing System.

[54] Guo J., Yun S., Meng Y., He N., Ye D., Zhao Z., Jia L., Yang L. (2023). Prediction of heating and cooling loads based on light gradient boosting machine algorithms. Build. Environ..

[55] Akiba T., Sano S., Yanase T., Ohta T., Koyama M. Optuna: A next-generation hyperparameter optimization framework. Proceedings of the 25th ACM SIGKDD International Conference on Knowledge Discovery & Data Mining (KDD ’19).

[56] R Core Team (2023). R: A Language and Environment for Statistical Computing.

[57] RStudio (2023). RStudio: Integrated Development for R..

[58] Wickham H. (2016). ggplot2: Elegant Graphics for Data Analysis.

[59] Hwang Y., Song J. (2023). Recent deep learning methods for tabular data. Commun. Stat. Appl. Methods.

[60] Schimleck L., Ma T., Inagaki T., Tsuchikawa S. (2023). Review of near infrared hyperspectral imaging applications related to wood and wood products. Appl. Spectrosc. Rev..

